# Bony Mallet Thumb Treated With the Ishiguro Extension Block Technique: A Case Report

**DOI:** 10.7759/cureus.54976

**Published:** 2024-02-26

**Authors:** Fuminori Kamakura, Gaku Yasuda, Yoshimasa Ishigaki, Satoshi Goto

**Affiliations:** 1 Department of Orthopedic Surgery, Fujimi-Kogen Hospital, Fujimi-Kogen Medical Center, Fujimi, JPN

**Keywords:** postoperative function of thumb, interphalangeal joint of thumb, ishiguro extension block technique, operative treatment, bony mallet thumb

## Abstract

A bony mallet thumb is an extremely rare injury. An 82-year-old man fell from a standing height and injured his right thumb. Imaging examinations revealed a rare intra-articular fracture at the dorsal side of the base of the distal phalanx of the thumb called the bony mallet thumb. Conservative treatment was adopted initially; however, surgery was deemed necessary because of the redislocation of the bone fragment. Thus, the Ishiguro extension block technique was used, and three months later, satisfactory thumb function was achieved. The Ishiguro technique is a relatively simple procedure often performed for bony mallet fingers. The current case indicated that it can also be used to treat cases of bony mallet thumbs successfully.

## Introduction

An avulsion fracture of the distal phalanx at the attachment part of the extensor tendon is called a bony mallet injury. Unlike mallet injuries of the index to little fingers, the bony mallet thumb is extremely rare [1-10]. Although some surgical and non-surgical procedures for a bony mallet thumb have been reported [2-9], the Ishiguro extension block technique shows promise for achieving good bone union and postoperative thumb function [1]. Herein, we present this rare injury and surgical technique with a literature review. This injury is extremely rare in clinical practice; therefore, it should be reported to aid clinicians’ decision-making regarding treatment.

## Case presentation

**Figure 1:**
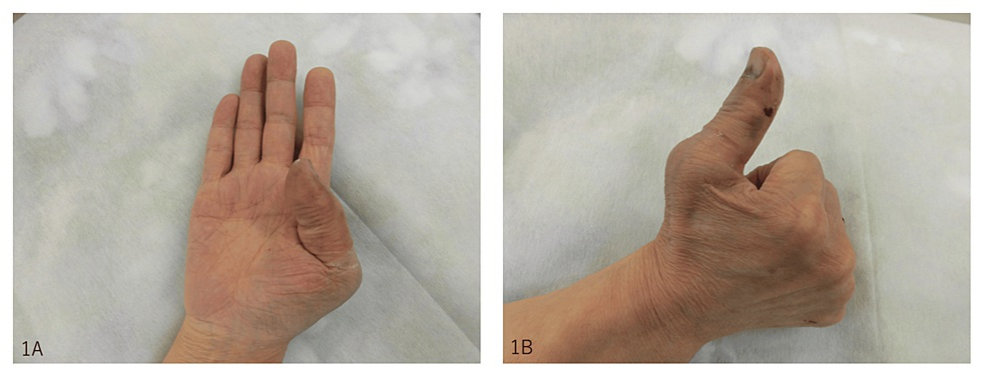
Before operation. The active range of motion (ROM) of the interphalangeal (IP) joint of the right thumb was 15° in flexion (1A) and 15° in extension (1B).

An 82-year-old man fell from a standing height and injured his right thumb. He presented to the orthopedic department of our hospital on the day of the injury. The thumb was swollen and painful, and the active range of motion (ROM) of the interphalangeal (IP) joint of the right thumb was 15° in flexion and 15° in extension. The disabilities of the arm, shoulder, and hand (DASH) score was 57.5/100 points (Figure 1A, 1B).

**Figure 2:**
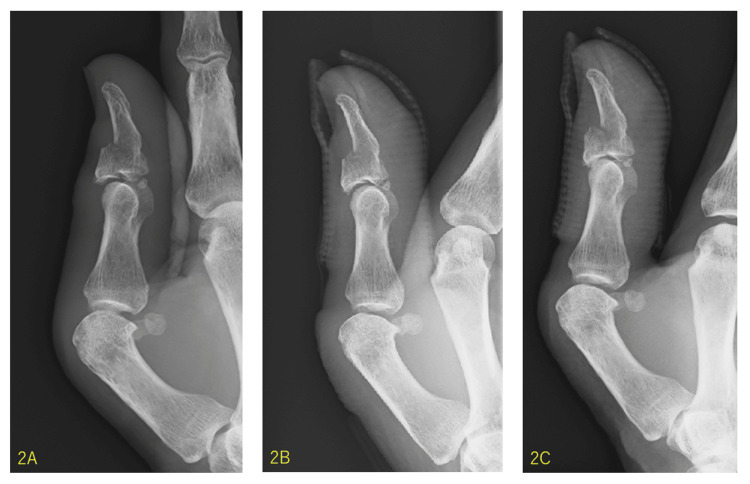
X-ray radiographs before operation. The X-ray radiography at the time of injury showed an intra-articular fracture at approximately 40% of the dorsal side of the base of the distal phalanx, with mild transposition (2A). In the X-ray radiography after the non-invasive reduction, the dorsal fragment was reduced non-invasively under the X-ray fluoroscope (2B). In the X-ray radiography two weeks after the non-invasive reduction, the dorsal fragment dislocated again (2C).

An X-ray radiograph showed an intra-articular fracture at approximately 40% of the dorsal side of the base of the distal phalanx, with mild transposition (Figure 2A). First, we reduced the fracture fragment non-invasively using fluoroscopy and obtained a successful reduction (Figure 2B). The IP joint was immobilized using a cast. However, two weeks after reduction, the dorsal fragment dislocated again (Figure 2C); therefore, we decided to perform surgery.

**Figure 3:**
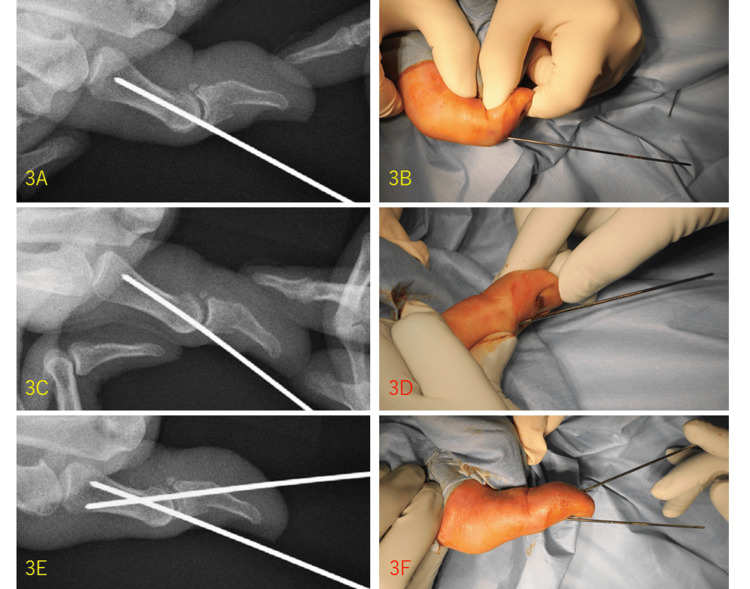
Intraoperative pictures of Ishiguro extension brock technique. One 1.5 mm K-wire was passed through close by above the dorsal fragment and introduced into the proximal phalanx under fluoroscopy (3A and 3B). The distal phalanx was pulled distally, and the IP joint was bent as dorsally as possible (3C and 3D). Another 1.5 mm K-wire was introduced from the volar aspect of the distal phalanx to the proximal phalanx (3E and 3F).

**Figure 4:**
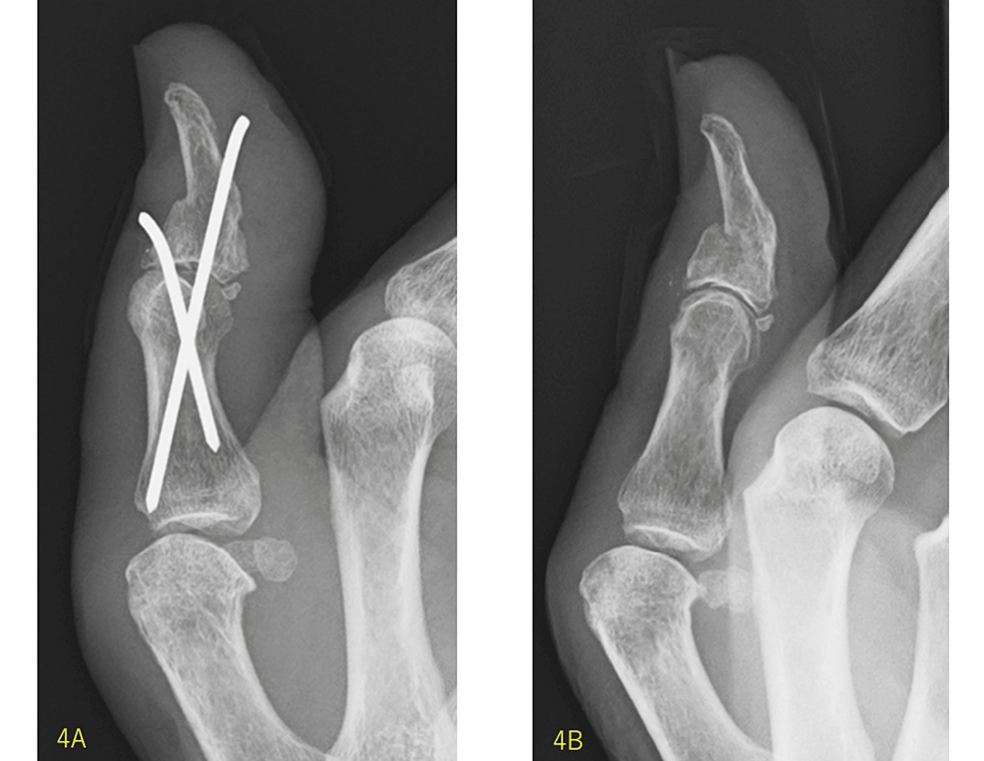
X-ray radiography after the operation. The Ishiguro extension block technique was carried out with two 1.5 mm K-wires. The fracture fragment was stabilized well. The K-wires were cut as short as possible and buried under the skin (4A). In the X-ray radiography six weeks after the operation, the K-wires were removed, and the bone union was good (4B).

The Ishiguro extension block technique was performed with two 1.5 mm Kirchner wires (K-wires), and the fracture fragment was well stabilized (Figures 3A-3F, 4A). The IP joint of the thumb was immobilized with a cast for six weeks, and the K-wires were removed with a good bone union (Figure 4B).

**Figure 5:**
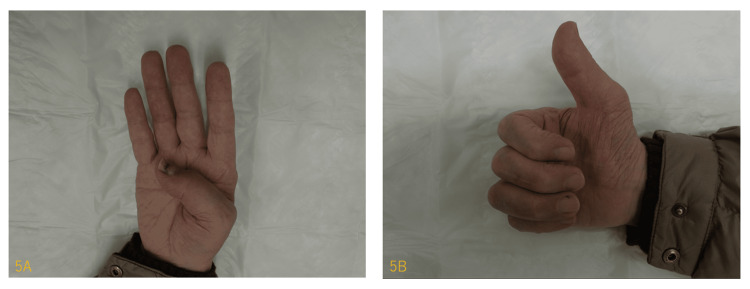
Three months after the operation. The active range of motion (ROM) of the interphalangeal (IP) joint was 25° in flexion (5A) and 15° in extension (5B). The patient was satisfied with the use of thumb in daily living.

At three months, the active ROM of the IP joint was 25° in flexion and 15° in extension. The DASH score was 20.8/100 points (Figure 5A, 5B). The patient was satisfied with the use of the thumb in daily living.

## Discussion

A bony mallet thumb is a very rare injury. Because the thumb has a short and greater form and the extensor pollicis longus (EPL) tendon has a thick and strong structure, a mallet (tendinous and bony) thumb is uncommon [9]. In most reported cases, the injuries were caused by relatively high energy, machinery, or sports, and in some cases, open injuries [2-9]. However, in the present case, the fracture was caused by a fall from the standing position, a low-energy accident. In bony mallet injuries, the patient sometimes cannot actively extend the injured joint because the extension mechanism collapses at the site of the avulsion fracture [1]. However, in the present case, the patient could actively extend the IP joint because of the larger size of the dorsal bone fragment [1]. Radiography, especially the lateral view, is essential for diagnosing bony mallet thumbs (Figure 2A). Compared with mallet fractures of other fingers, a bony mallet thumb has no tendency for IP joint subluxation because of the tightness of the joint capsule [3]; no subluxation was found in the present case. Conservative treatment for a bony mallet thumb can also be considered, as demonstrated by some studies [4,5]; however, evidence is lacking. Regarding the surgical treatment for bony mallet injuries, including other fingers, direct pinning, extension block K-wire with direct pinning, compression pins, pullout wires, mini plates, hook plates, mini screws, tension band wiring, K-wire and suture, screw and suture, biodegradable implants [6], single extension block K-wire [7], nail plates [8], and two extension block K-wires [9], were indicated by previous studies. In the present case, we selected the Ishiguro extension block technique, which is generally used for bony mallet injuries of the other fingers [1].

The Ishiguro technique needs two K-wires. One is used as a dorsal block pin to stabilize the avulsion fragment, and the other is used for IP joint fixation. In our procedure, first, under digital block anesthesia, the IP joint was held in the maximum flexion position. Second, one 1.5 mm K-wire was passed close to the dorsal fragment and introduced into the proximal phalanx under fluoroscopy (Figures 3A, 3B). Third, the distal phalanx was pulled distally, and the IP joint was bent dorsally as much as possible (Figures 3C, 3D). Fourth, another 1.5 mm K-wire was introduced from the volar aspect of the distal phalanx to the proximal phalanx (Figures 3E, 3F). Finally, the K-wires were bent near the skin, cut, and buried under the skin, and the wound was stitched (Figure 4A). The IP joint of the thumb was immobilized using a cast in the extension position. As this technique does not require direct insertion of the K-wire into the fragment, there is no concern about breaking the bone. K-wires are usually removed after four weeks [1]. We continued immobilization with a cast for six weeks, considering the strong features of the EPL tendon, as mentioned above. As a new method, we cut the K-wires as short as possible and buried them under the skin, which enabled postoperative casting of the thumb, unlike splinting only in previous studies [6].

Osteoarthritic joint changes, delayed bone union, redislocations, joint contractures, nail deformities, skin problems, and infections have been reported as complications of K-wire techniques [1,6,10]. Although a higher incidence of local infection using the K-wire technique compared with the open technique has been reported [6], no infection occurred in the present case. This might have been influenced by the burial of the K-wires and the closure of the skin during the operation.

During postoperative rehabilitation, passive extension exercise is vital to alleviate contracture once the K-wires are removed. Active extension motion is allowed when there is no or only mild tendon injury [1]. Because open reduction methods can lead to worse functional outcomes of the thumb [6], closed K-wire methods may be beneficial if infection risk is avoided. In the present case, good postoperative function was achieved by using the Ishiguro extension block technique. This may be a good choice for treating bony mallet thumbs.

## Conclusions

We encountered an extremely rare case of a bony mallet thumb, successfully treated using the Ishiguro extension block technique. It may be difficult to achieve good results with conservative therapy as a treatment for injury. Considering the poor functional outcomes of open surgery, closed K-wire techniques may be preferable if infectious complications can be avoided. The Ishiguro extension block technique is a good option for treating bony mallet thumb injuries.
